# Low Density Lipoprotein Receptor-Related Protein-1 in Cardiac Inflammation and Infarct Healing

**DOI:** 10.3389/fcvm.2019.00051

**Published:** 2019-04-26

**Authors:** Nicola Potere, Marco Giuseppe Del Buono, Adolfo Gabriele Mauro, Antonio Abbate, Stefano Toldo

**Affiliations:** ^1^VCU Pauley Heart Center, Virginia Commonwealth University, Richmond, VA, United States; ^2^Department of Cardiovascular and Thoracic Sciences, Catholic University of the Sacred Heart, Rome, Italy

**Keywords:** low-density lipoprotein receptor-related protein-1, LRP1, acute myocardial infarction, ischemia-reperfusion, inflammation, cardioprotection, cardiac repair

## Abstract

Acute myocardial infarction (AMI) leads to myocardial cell death and ensuing sterile inflammatory response, which represents an attempt to clear cellular debris and promote cardiac repair. However, an overwhelming, unopposed or unresolved inflammatory response following AMI leads to further injury, worse remodeling and heart failure (HF). Additional therapies are therefore warranted to blunt the inflammatory response associated with ischemia and reperfusion and prevent long-term adverse events. Low-density lipoprotein receptor-related protein 1 (LRP1) is a ubiquitous endocytic cell surface receptor with the ability to recognize a wide range of structurally and functionally diverse ligands. LRP1 transduces multiple intracellular signal pathways regulating the inflammatory reaction, tissue remodeling and cell survival after organ injury. In preclinical studies, activation of LRP1-mediated signaling in the heart with non-selective and selective LRP1 agonists is linked with a powerful cardioprotective effect, reducing infarct size and cardiac dysfunction after AMI. The data from early phase clinical studies with plasma-derived α1-antitrypsin (AAT), an endogenous LRP1 agonist, and SP16 peptide, a synthetic LRP1 agonist, support the translational value of LRP1 as a novel therapeutic target in AMI. In this review, we will summarize the cellular and molecular bases of LRP1 functions in modulating the inflammatory reaction and the reparative process after injury in various peripheral tissues, and discuss recent evidences implicating LRP1 in myocardial inflammation and infarct healing.

## Introduction

Despite therapeutic advances, acute myocardial infarction (AMI) remains the most common cause of heart failure (HF) worldwide and is associated with an unacceptable high rate of morbidity and mortality (1) AMI is often the result of an abrupt destabilization of a coronary atherosclerotic plaque with superimposed thrombosis that leads to prolonged perturbation of oxygen delivery to the heart, causing myocardial cell death (2). The introduction of primary percutaneous coronary intervention (PCI) and the optimization of medical therapy drastically changed the treatment and improved the prognosis associated with AMI (3). The prompt restoration of coronary blood flow in the occluded artery salvages a large amount of myocardium at risk in a time-dependent fashion reducing the infarct size (“*time is muscle*”). However, the reperfusion of acutely ischemic myocardium independently induces myocardial damage, significantly contributing to determine the final infarct size due to ischemia-reperfusion injury (IRI) (4, 5). IRI is a complex multifaceted process of regulated and unregulated cell death associated with a strong inflammatory reaction, neurohumoral activation, and oxidative stress that overall leads to paradoxical cardiomyocyte dysfunction and tissue damage, failing to salvage all the viable ischemic myocardium (4–8). It is estimated that strategies aimed at limiting IRI in AMI may reduce the infarct size by a further 25%, thus maximizing the benefits of reperfusion (4). This is of outmost importance because the size of the infarct (initial damage) is a predictor of clinical outcome and 30% of patients after AMI undergo detrimental structural and functional changes in the area bordering the infarct and in the remote viable remote myocardium (“adverse left ventricular [LV] remodeling”), with negative clinical and prognostic implications (9, 10). Over the last decades, together with improving timing and technical reperfusion strategies and pharmacological neuro-hormonal blockade, great interest was drawn in understanding the pathophysiological mechanisms driving IRI and its contribution to adverse LV remodeling, leading to a large volume of experimental preclinical and clinical studies (4–8, 11, 12). It has also become clear that the infarct size, albeit the most important, is not the only predictor of progressive adverse LV remodeling. A delicate interplay between infarct size, exuberant acute and chronic persistent inflammation, increased wall stress and neurohormonal activation has been described, and these factors synergistically contribute to determine the progressive deterioration of the ventricle structure and function after AMI (10).

### Inflammation After AMI: From Healing Response to Mechanism of Disease

Inflammation, triggered by ischemia (initial damage) and amplified by the reperfusion (additional damage), is an essential and active component of tissue healing following injury as it is useful to remove necrotic cell debris in the damaged myocardium (6, 13, 14). The initial inflammatory response following the ischemia and reperfusion is then followed by an anti-inflammatory reparative phase leading to myocardial scar formation (14). This is a finely regulated process, in which interactions between immune soluble mediators and immune cells, as well as cardiac resident cells and local and systemic humoral factors, coordinate the healing process in a timely manner. Perturbation in this delicate balance may lead to a prolonged inflammatory state that acutely worsens the infarct size and chronically contributes to post-MI adverse LV remodeling (6, 14–17).

The inflammatory response is initiated by myocardial necrosis and damaged extracellular matrix (ECM) exposing endogenous alarm signals (damage-associated molecular patterns [DAMPs]) and activating components of the complement system that work as ligands for pattern recognition receptors (PRRs). PPRs include toll-like receptors (TLRs) and inflammasomes (13, 15). TLRs initiates multiple inflammatory cascades mediated by nuclear factor-kappa B (NF-κB), and “prime” the cells by increasing the transcription of several proteins and cytokines, including those that take part to the inflammasome pathway (13, 15). This process takes place in different cellular lines (i.e., cardiac resident cells and circulating leukocytes) in a cell-type specific fashion and is amplified by reactive oxygen species (ROS) production that originate at the moment of ischemia and reperfusion and continue to be produced during the acute phase of inflammation (14). The inflammasome pathway is implicated in the development of coronary artery disease and growth of the infarct size post-AMI (6, 13, 15). Inflammasomes are a group of macromolecular protein complexes that regulate the local and systemic response to pathogen invasion and/or tissue damage. The NACHT, LRR, and PYD domains-containing protein 3 (NLRP3), is the better characterized inflammasome receptor in the heart because of its involvement in response to cardiac injury (13, 15). The inflammatory priming initiated by DAMPS and PRRs as well as TLRs induces a robust increase in the inflammasome components. In primed cells, a second signal, termed as “trigger” and mainly dependent on intracellular potassium concentration, activates NLRP3 (13, 15). The active NLRP3 oligomerizes, forming a structure that drives the polymerization of apoptosis-associated speck-like protein containing a CARD (ASC), which in turn functions as a central core for caspase-1 recruitment and activation, resulting in (1) the cleavage of the inactive pro-IL-1β and pro-IL-18 in their active forms IL-1β and IL-18, and in (2) a form of pro-inflammatory programmed cell-death known as pyroptosis (6, 13, 15). IRI creates the ideal conditions for both priming and triggering the inflammasome, whose expression increases overtime reaching the higher levels after 1–3 h from ischemia-reperfusion, thus contributing to exacerbate the inflammatory reaction (6, 13, 18). The production of mature IL-1β and IL-18 induces autocrine, paracrine, and endocrine signaling cascades involving myeloid differentiation primary response 88 (MYD88) and NF-κB, resulting in recruitment of immune cells (neutrophils, monocytes, macrophages, and lymphocytes) to the site of injury, increase in chemokine concentration and depression of contractile function (6, 13, 18).

Once the inflammatory response to tissue injury has taken place, several days after the AMI, changes in cellular environment through the expression of cytokines involved in resolution of inflammation (especially IL-10 and transforming growth factor (TGF)-β1 occurs. Neutrophils are replaced by macrophages with pro-angiogenic and pro-fibrotic phenotypes, fibroblasts and lymphocytes that are necessary for resolution of inflammation and subsequent scar formation (10, 14). The acute drop in cardiac output results in an adaption of volume, shape, structure, and/or function of the heart that rescues the hemodynamics from collapse. However, the increased wall stress, neurohormonal activation, sympathetic activation, and chronic inflammation (as documented by the persistent increase in C-reactive protein, a systemic surrogate for IL-1 activity) worsen the cardiac remodeling process after injury, and depress myocardial function leading to adverse cardiac remodeling and HF (10, 14). With a deeper understanding of the role of inflammation after AMI, it has become clear that some of the previously proposed anti-inflammatory strategies are ineffective or not recommended following AMI (17). Novel therapeutic targets are therefore warranted to reduce the inflammatory damage associated with AMI, thus limiting infarct size and preventing long-term adverse effects (16, 18). Among these, the multiligand, multifunctional and ubiquitous receptor, low-density lipoprotein receptor related protein-1 (LRP1), has emerged as a modulator of tissue inflammation and repair in several organs including the brain, lung, kidney and vasculature (19). Interestingly, recent research demonstrated that LRP1 also plays a major role in AMI (20, 21).

In the present review, we summarize some cellular and molecular aspects of LRP1 endocytic/signaling functions in orchestrating the inflammatory response and in balancing tissue repair, and discuss the role of LRP1 in the pathobiology of IRI and infarct healing. We will then describe the current efforts to therapeutically target LRP1 upon reperfusion to quench the inflammatory response after AMI, thus limiting infarct size and preventing adverse LV remodeling and HF.

## Low-Density Lipoprotein Receptor-Related Protein 1 (LRP1): General Features

**Figure 1:**
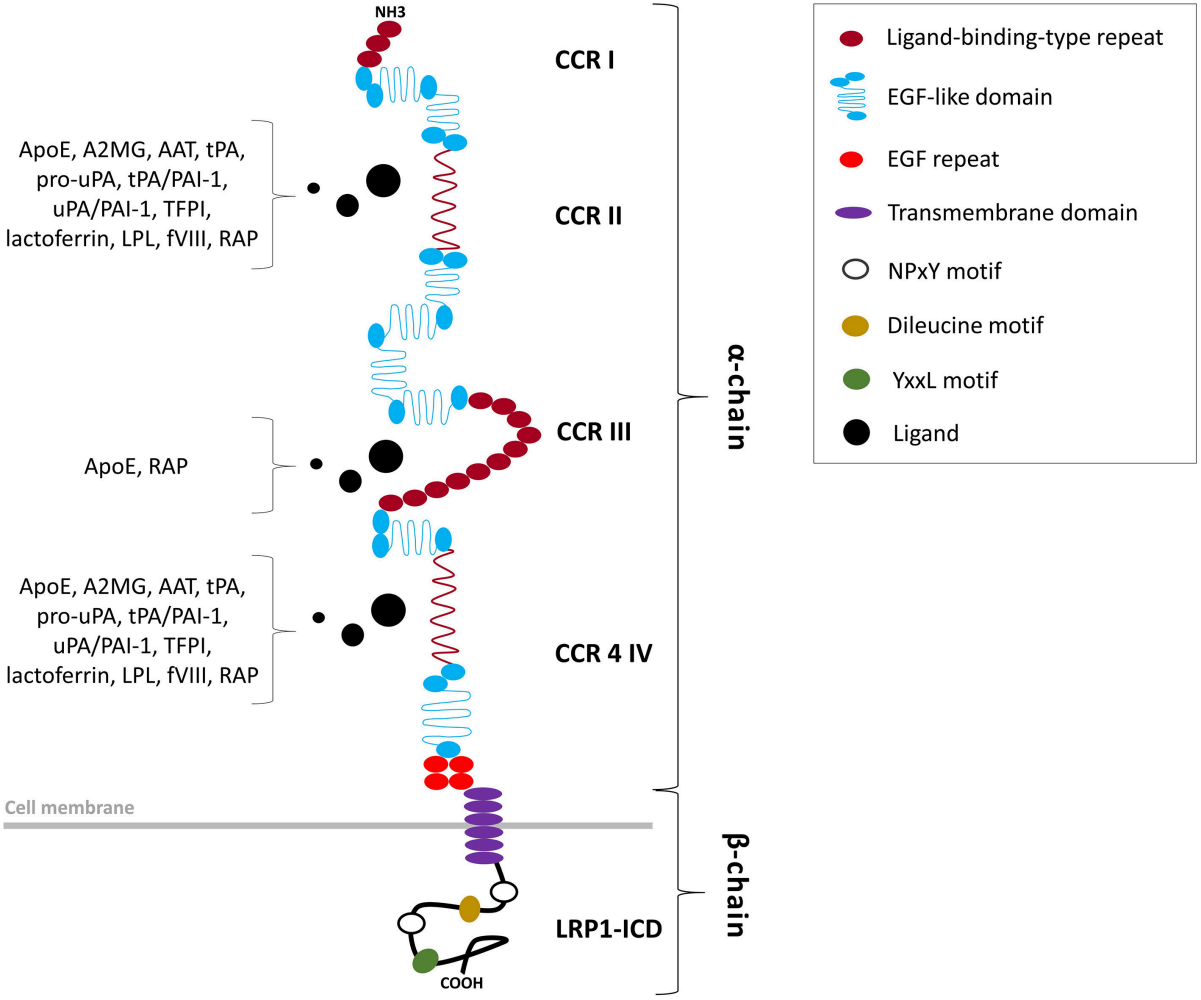
Structural organization of the LRP1 receptor. LRP1 is a type 1 transmembrane receptor consisting of a 515-kDa entirely extracellular α-chain non-covalently bound to an intracellular 85-kDa β-chain. The α-chain, primarily responsible of the ligand-binding activity of LRP1, includes four clusters of complement-like repeats (CCRs I-IV) and EGF-like domains. The β-chain includes a tetra amino acidic YxxL motif, two NPxY motifs, which serve as docking sites for signaling adapter proteins, and numerous tyrosine residues, whose phosphorylation is necessary for LRP1-mediated signal transduction.

**Figure 2:**
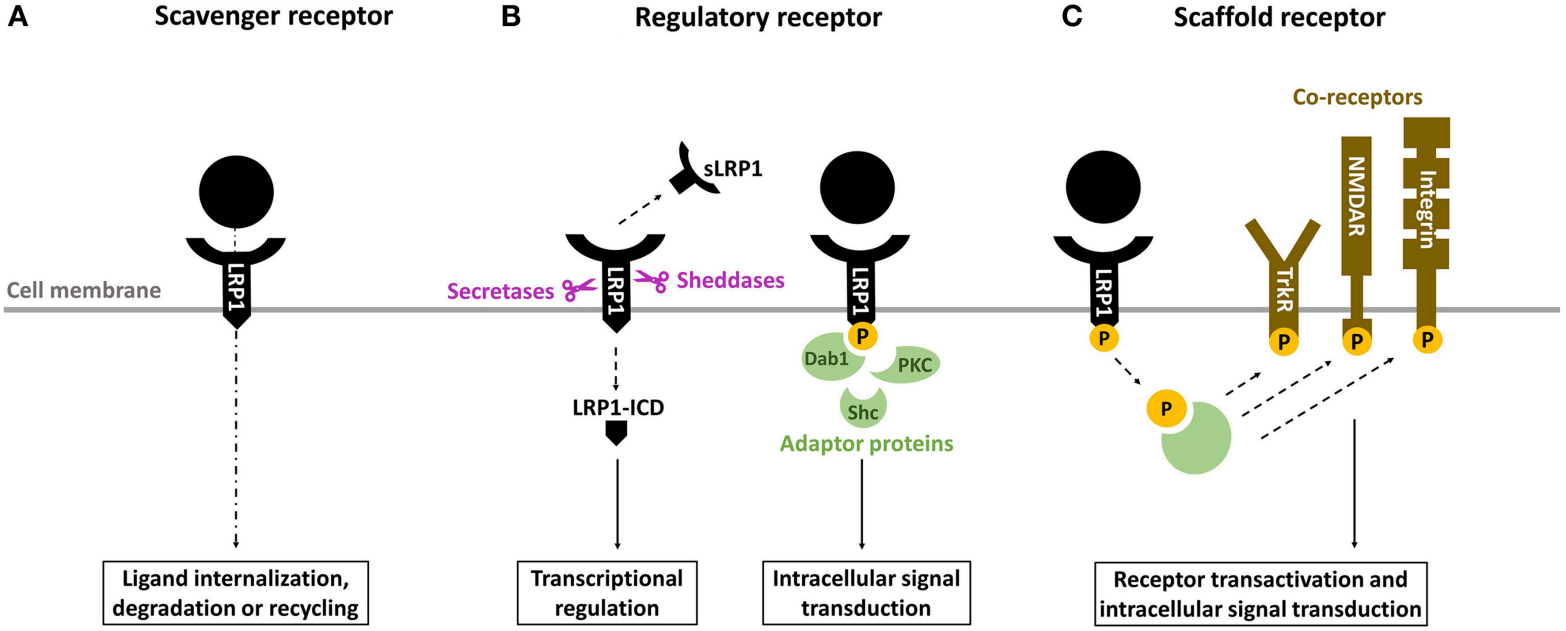
Overview of the biological properties of LRP1. **(A)** as a scavenger receptor, LRP1 binds and internalizes a wide range of ligands for degradation in lysosomes or recycling. **(B)** as a regulatory receptor, LRP1 modulates gene transcription through its intracellular domain (LRP-ICD) generated by intramembrane proteolysis and releases its extracellular ectodomain (soluble LRP [sLRP1]) in the circulation, where it exerts numerous biological functions. Alternatively, LRP1-ICD has the ability to recruit various adaptor proteins to transduce multiple intracellular signals. **(C)** as a scaffold receptor, LRP1 modulates the activity of other membrane proteins including integrins, tyrosine kinase (Trk) receptors and N-methyl-D-aspartate (NMDA) receptors.

**Figure 3:**
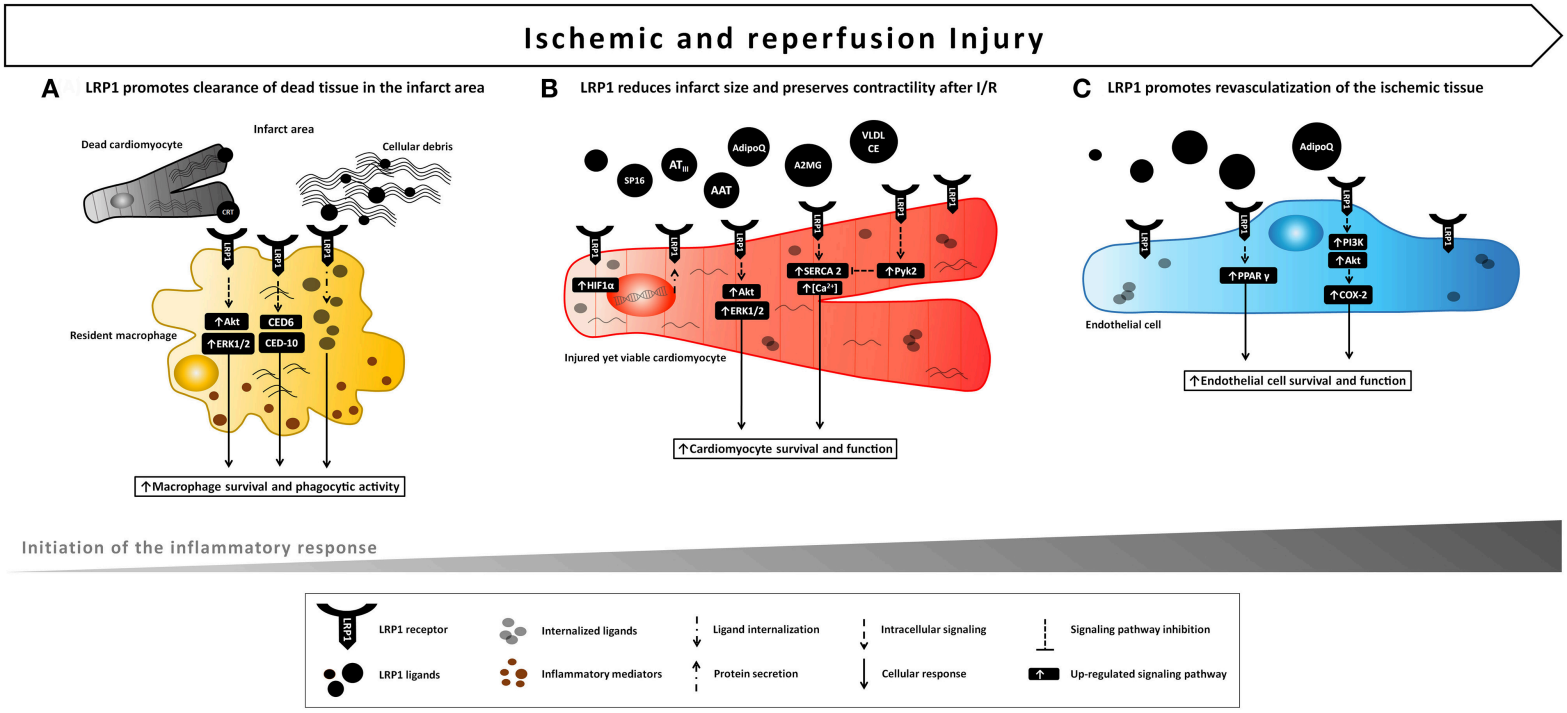
Proposed model of LRP1 involvement in ischemic e reperfusion injury. LRP1 and its ligands are markedly up-regulated in the myocardial tissue following AMI. **(A)** LRP1 promotes macrophage survival and phagocytic activity, thus facilitating clearance of cellular debris and apoptotic bodies and removing potentially dangerous byproducts after the ischemic injury. **(B)** Binding of certain ligands including serine protease inhibitors (AAT, A2MG, AT_III_) to LRP1 leads to a cardioprotective signal reducing infarct size and preservation of cardiac systolic function following acute ischemia and reperfusion. A comparable cardioprotective effect can be leveraged by the use of a small synthetic peptide, SP16, functioning as LRP1 agonist. **(C)** Activation of LPR1-mediated signaling induces endothelial cell migration, differentiation and survival leading to improved endothelial cell function and revascularization of ischemic tissue.

LRP1, or cluster of differentiation 91 (CD91), is a transmembrane receptor that has long been known for its role in lipoprotein endocytosis (19) (Figure 1). It is now recognized, however, that LRP1 is a multifunctional protein: (1) as a scavenger receptor, LRP1 internalizes a plethora of extracellular ligands (Table 1) (Figure 2A); (2) as a regulatory receptor, it modulates cellular signaling in response to various extracellular stimuli (Figure 2B); (3) as a scaffold receptor, LRP1 has also the ability to partner with, and modulate, the activity of other membrane proteins such as integrins and receptor tyrosine kinases (19, 22–24) (Figure 2C). These peculiar properties enable LRP1 to couple extracellular microenvironment and intracellular signaling and response.

**Table 1:** LRP1 ligands.

**Proteins involved in lipoprotein metabolism**
Apo E and apo E-enriched lipoproteins (VLDL and chylomicron remnants), lipoprotein lipase, hepatic lipase
**Proteases and protease/inhibitor complexes**
A2MG and A2MG/protease complexes; tPA, uPA, tPA/PAI-1 and uPA/PAI-1 complexes; AAT and AAT/elastase complexes; AT_III_ and thrombin/AT_III_ complexes;
MMP-2, MMP-9, MMP-13 (free or complexed with TIMPs); TFPI and factor VIIa/TFPI, fVIIIa, fIXa, and fIXa/protease nexin-1;
**Matrix proteins**
Thrombospondin-1, thrombospondin-2, fibronectin;
**Growth factors**
PDGF, TGF-β, CTGF;
**Others**
Calreticulin, collectins (via calreticulin), lactoferrin, complement C1 and C3, RAP;

*Apo E, apolipoprotein E; VLDL, very low density lipoprotein; A2MG, α2-macroglobulin; tPA, tissue-type plasminogen activator; uPA, urokinase plasminogen activator; PAI-1, plasminogen activator inhibitor 1; AT_III_, antithrombin III; MMP, metalloproteinase; TIMPs, tissue inhibitors of metalloproteinases; TFPI, tissue factor pathway inhibition; PDGF, platelet-derived growth factor; TGF-β, transforming growth factor β; CTGF, connective tissue growth factor; RAP, receptor associated protein*.

LRP1 is a ubiquitous protein, abundantly expressed in the liver, brain, kidney, lung, vasculatures, and in the heart after injury (20, 22–25). The importance of LRP1 is demonstrated by the lethality of Lrp1 gene deletion as it arrests mouse embryo development at an early stage of development (26). The endocytic function and signaling properties confer a major role to LRP1 in the pathophysiology of numerous diseases such as hepatic steatosis, kidney fibrosis, acute respiratory distress syndrome (ARDS), Alzheimer's disease (AD) and atherosclerosis (23). In addition, recent evidences showed that LRP1 is implicated in the pathophysiological mechanisms underlying AMI, IRI and adverse LV remodeling (20, 21).

The LRP1 receptor belongs to the LDLR superfamily and structurally consists of five domains (Figure 1). The mature two-chain structure of LRP1 is generated by furin-like endoproteases from a 600-kDa precursor in the trans-Golgi compartment (19, 22). The entirely extracellular 515-kDa α-chain consists of four clusters of complement-like repeats (CCRs), functioning as ligand-binding sites, separated by epidermal growth factor (EGF) repeats. The transmembrane 85-kDa β-chain, non-covalently conjugated to the former, consists of YxxL and dileucine motifs, serving as principal endocytosis signals, and two NPxY motifs, functioning as secondary endocytosis signals and docking sites for cytoplasmic adaptor proteins such as Disabled-1, protein kinase α (PKCα), Shc and FE65, which mediate LRP1-dependent signal transduction (19, 22, 27) (Table 2). LRP1 initiates signaling by direct ligand binding or, through its co-receptors, transactivates signal pathways. Although the exact mechanisms have been partly elucidated, the tyrosine phosphorylation at the NPxY motifs is necessary for LRP1-mediated signal transduction (21, 23, 27).

**Table 2:** Adaptor proteins known to bind to the cytoplasmic domain of LRP1.

**Adaptor protein**	**Function**
Dab1	Activation of downstream Src kinases; regulation of neurogenesis and neuronal motility
PKCα	Regulation of apoptosis, inflammation, proliferation, differentiation and motility
Shc	Activation of downstream tyrosine kinases
JIP-1, JIP-2	Activation of downstream MAPK kinases
GULP	Regulation of phagocytosis
PSD95	Coupling to NMDA receptors
FE65	Regulation of actin dynamics, APP processing, neuronal growth and migration

*Dab1, disabled-1, PKCα, protein kinase C α; JIP, JNK (c-Jun N-terminal kinase)-interacting proteins; MAPK, mitogen-activated protein kinase; PSD95, post-synaptic density protein 95; NMDA, N-methyl-D-aspartate receptor*.

LRP1 interacts with more than 100 ligands, including proteins involved in lipoprotein metabolism such as apolipoprotein E (ApoE) and very low-density lipoprotein (VLDL), activated coagulation factors, growth factors, matrix proteins, proteinases and proteinase-inhibitor complexes (19) (Table 1). The serine proteinase inhibitors (SERPINs) are a wide family of proteins that forms complexes with target plasma proteinases and modulate their concentration/activity (28). However, the resulting SERPIN-enzyme complexes (SECs) are chemically unstable and would dissociate over time, releasing the active enzymes in the circulation (28). LRP1, also referred to as SEC receptor, has the ability to recognize the entire spectrum of SERPINs including α1-antitripsin (AAT), α2-macroglobulin (A2MG) and antithrombin III (AT_III_), and mediate the endocytosis of these complexes for intracellular degradation (24, 28). Interestingly, certain ligands, such as SERPINs and others, bind to LRP1, and functioning as LRP1 agonists, have been shown to stimulate LRP1 signaling that regulates multiple biological processes in several organs including the heart (21, 24, 28, 120–124).

We will hereafter discuss the anti-inflammatory roles of LRP1-mediated signaling in both the innate and adaptive immune responses, and in tissue repair and remodeling after injury.

### LRP1 as a Modulator of the Innate Immune Response

The maintenance of tissue homeostasis necessitates both the recognition and removal of invading microbial pathogens as well as the clearance of dying cells (29). Phagocytosis is an essential component of the innate immune response and is referred to as engulfment and destruction of invading microorganisms (29). Phagocytosis is also required for the clearance of apoptotic bodies and cellular debris following injury. Dysregulation of phagocytosis has been linked to the pathogenesis of several chronic diseases (29). During microbial infections, LRP1 plays a dual role in pathogen clearance by scavenging bacterial membrane lipoproteins, and local control of the inflammatory reaction by preventing an exaggerated inflammatory response leading to further injury (30).

Early studies identified LRP1 as a receptor involved in the phagocytosis of apoptotic cells in mammals (31). LRP1 interacted with the cytoplasmic adaptor protein engulfment adapter protein (CED-6/GULP1), that initiates phagocytosis by activating CED-10, a rac GTPase necessary in the reorganization of the cytoskeleton during phagocytosis (31) Tissue-resident macrophages represent the first line of defense and their primary function is to clear potential noxious agents (bacteria, apoptotic or necrotic cells, irritants), which may potentially trigger the inflammatory response. Macrophages transfected with chimeric proteins containing LRP1 cytoplasmic tails and heterologous extracellular domains capable of binding non-opsonized sheep red blood cells (SRBCs) were able to internalize SRBCs (32). The LRP1 receptor required calreticulin (CRT) and members of the collectin family of proteins for an efficient clearance of apoptotic bodies. The binding of the complex LRP1/CRT to apoptotic cells opsonized with complement 1q (C1q) or mannose-binding lecitin (MBL) was shown to trigger phagocytosis whereas blockage of LRP1 reduced apoptotic cell clearance by resident alveolar macrophages (rAM) (33). The LRP1/CRT complex on rAM was also shown to recognize surfactant proteins (SPs), a specialized group of alveolar collectins, bound on target material, stimulate phagocytosis and modulate the ensuing release of inflammatory mediators (34, 35). Alternatively, LRP1 directly recognized CRT on the surface of non-opsonized apoptotic cells to mediate phagocytosis (36). Cell surface CRT on viable or apoptotic neurons interacted with microglial LRP1 to induce phagocytosis (37). In primary cultures of rat microglia, astrocytes and oligodendrocytes, LRP1 mediated the uptake and subsequent accumulation in lysosomes of myelin vescicles (MV), which were blocked by LRP1 antagonism or gene-silencing (38). LRP1 protein expression was substantially increased in the cerebellum and in the spinal cord after experimental autoimmune encephalomyelitis in mouse, an experimental model of multiple sclerosis, suggesting that LRP1 is major receptor for phagocytosis of degraded myelin (38). Moreover, LRP1 deficiency reduced Akt survival pathway activation resulting in increased macrophage apoptosis, whereas macrophages expressing LRP1 were more likely to phagocytize other dying cells in the atherosclerotic plaque, thus limiting necrotic core formation (39).

Therefore, by promoting phagocytosis of pathogens, apoptotic bodies and cellular debris, LRP1 removes potentially dangerous materials, and orchestrates the initiation of the adaptive immune response in different organs and diseases (Figure 3A).

### LRP1 as a Modulator of the Adaptive Immune Response

**Figure 4:**
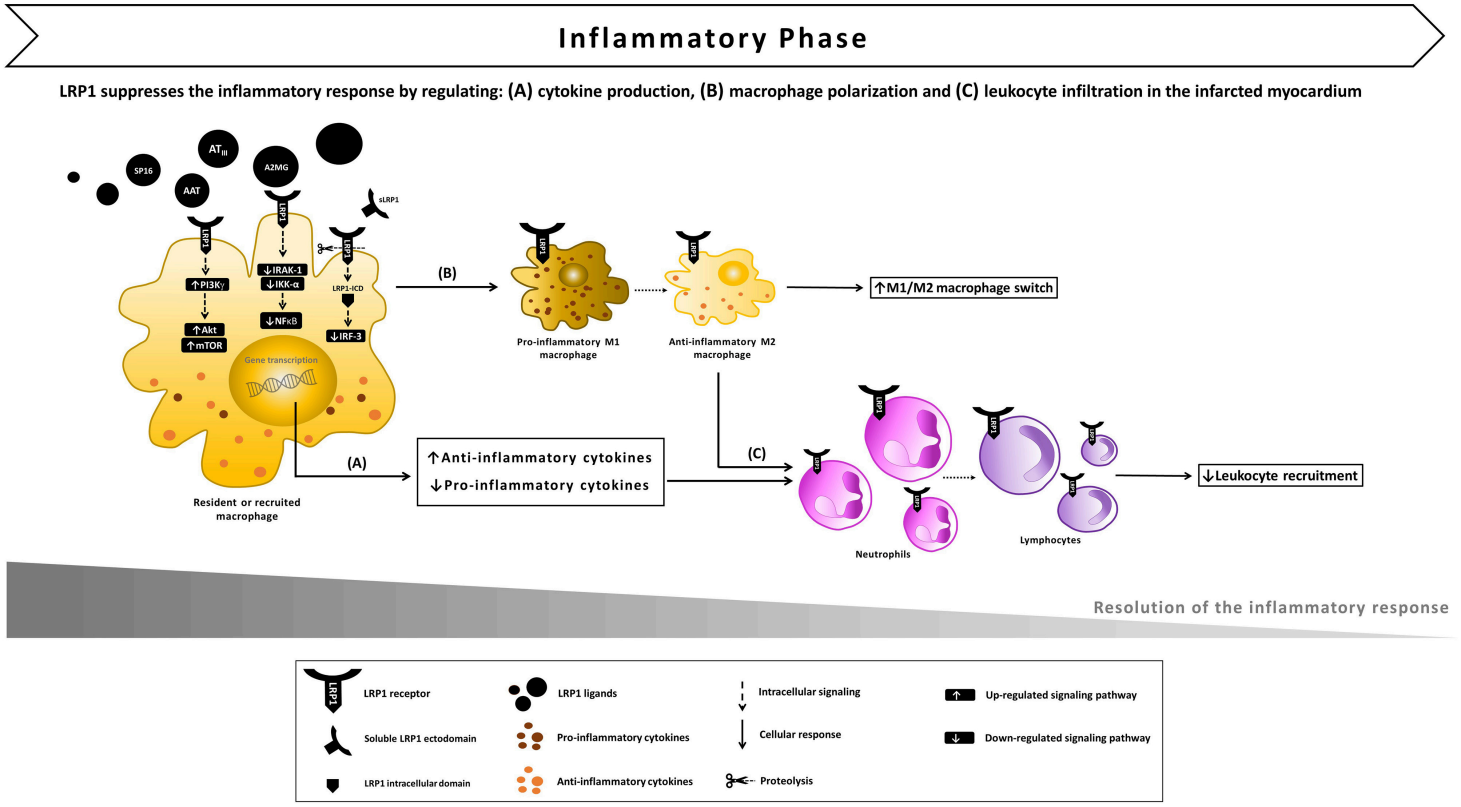
Proposed model of LRP1 involvement in the inflammatory phase following AMI. **(A)** After ligand binding, LRP1 undergoes proteolysis by γ-secretases, the intracellular domain (LRP-ICD) migrates to the nucleus binding to the interferon regulatory factor-3 (IRF-3) and promoting IRF-3 nuclear export and proteasomal degradation, thus reducing the expression of pro-inflammatory genes. The LRP1-agonist complex also inhibits the interleukin-1 receptor associated kinase-1 (IRAK-1), leading to an inhibitory signal on nuclear factor-kappa B (NF-κB). In addition, multiple toll-like receptors (TLRs) have the ability to activate LRP1 which, in turn, stimulates downstream Akt/mTOR signaling which biases cytokine production outputs and restrains the inflammatory response. **(B)** LRP1 also appears to facilitate the M1/M2 macrophage conversion, promoting the development of an anti-inflammatory M2 functional phenotype. **(C)** As a result of LRP1-induced anti-inflammatory signaling, leukocyte infiltration in the infarcted myocardium is reduced.

Inflammation is a beneficial, adaptive immune response when triggered by infection or tissue injury (40). Following the recognition of noxious material by phagocytic cells, the rapid activation of a complex signaling network leads to the release of critical pro-inflammatory mediators and the recruitment of immune cells to the site of injury. A regulated inflammatory response ultimately leads to the containment of the noxious agents and the restoration of tissue integrity. However, dysregulated or excessive inflammation leads to greater injury and prevents tissue healing, thus contributing to disease pathogenesis in cardiovascular, kidney, lung, autoimmune, skeletal, muscular and neurodegenerative diseases (40). Identification of novel pro- and anti-inflammatory factors and pathways remains an important challenge for addressing these disease states. Recent research has shown that LRP1 modulates several of these inflammatory pathways (30, 41) (Figure 4).

#### LRP1 in the Response to Lipopolysaccharide (LPS)

LPS is an important structural component of the outer membrane of Gram-negative bacteria. Binding of LPS to the cell surface of TLRs, including TLR4, induces a massive inflammatory response that may potentially cause acute sepsis or chronic inflammatory disorders if excessive stimulation occurs (42). Upon LPS recognition, TLR4 activates a complex intracellular signaling network ultimately resulting in the translocation of the transcription factors interferon regulatory factor 3 (IRF-3) and NF-κB to the nucleus, and the expression of pro-inflammatory genes (42). Various studies revealed that LRP1 limits the LPS-induced inflammatory response in multiple cell types and experimental models of injury (30, 41).

In macrophages, LPS-TLR4 interaction culminated in the γ-secretase-mediated sequential cleavages ultimately leading to the release of LRP1 intracellular domain (LRP1-ICD) and its translocation to the nucleus (43). In the nucleus, LRP1-ICD regulated gene expression by binding and facilitating nuclear export of IRF-3, thus repressing the expression of pro-inflammatory genes (43). This negative-feedback loop indicates that LRP1 exerts an anti-inflammatory effect in macrophages when exposed to LPS. Furthermore, LPS-activated TLRs (TLR2/6, 3, 4, and 9) promoted the activity of LRP1 in human and mouse primary macrophages and induced its phosphorylation at Y4507 (44). LRP1 phosphorylation induced the formation of a ternary complex, which coincided on micropinosomal membranes, with the guanosine triphosphatase (GTPase) Rab 8a and phosphatidylinositol 3-kinase γ (PI3Kγ) (44). This effector complex, in turn, activated Akt/mTOR downstream signaling to reprogram macrophages and bias inflammatory cytokine production. CRISPR-mediated knockout of LRP1 in macrophages altered Akt/mTOR signaling, resulting in enhanced synthesis of IL-6 and IL-12 and reduced output of the regulatory cytokine IL-10 compared to control cells (44) (Figure 4A). Thus, the LPS-TLRs-LRP1 axis represents a key mechanism through which LRP1 helps suppress inflammation in peripheral tissues.

In a murine model of atherosclerosis, macrophage-specific LRP1 deletion enhanced atherosclerotic plaque development and intralesional inflammation and cellularity (45). Similarly, loss of LRP1 on vascular smooth muscle cells (VSMCs) resulted in a dramatic increase in atherosclerosis (46). Binding of apolipoprotein E (ApoE) to VSMC LRP1 was found to target interleukin-1 receptor associated kinase-1 (IRAK-1) activation and down-regulate the pro-inflammatory transcription factor NF-κB, thus interrupting the IL-1β and IL-18 signaling (47). Moreover, LRP1 may also exert anti-atherogenic effects through mechanisms independent from ApoE, involving macrophage apoptosis and monocyte recruitment (48).

Mounting evidence demonstrated the involvement of LRP1 signaling in neuroinflammation, a condition characterized by microglial activation and increased levels of cytokines and chemokines in the central nervous system (CNS) (49). In microglial cells, ApoE-mediated LRP1 activation, attenuated LPS-induced inflammatory response by suppressing the c-Jun N-terminal kinase (JNK) pathway (50). Likewise, inhibition through its antagonist, receptor-associated protein (RAP), or deletion of LRP1, through tissue-specific LoxP/Cre recombination techniques, resulted in the activation of both JNK and NF-κB pathways with corresponding increased sensitivity to LPS in the production of pro-inflammatory cytokines (51). LRP1 was also observed to mediate the neuroprotective effects of astaxanthin (ATX), a natural carotenoid, by inhibiting, through JNK and NF-κB pathways suppression, inflammation and reversing LPS-induced M1/M2 polarization of microglial cells (52). Moreover, deletion of microglial LRP1 also negatively impacted the progression of mouse autoimmune encephalitis (53). As LRP1 is highly expressed in a variety of cell types in the CNS including glial cells, neurons and vascular cells, and serves as a major receptor for a wide range of molecules such as ApoE and amyloid β (Aβ), which play critical roles in AD, numerous studies addressed LRP1 endocytic/signaling functions for novel diagnostic and therapeutic strategies (54). These evidences suggest that the function of LRP1 in immune cells is to keep these cells in an anti-inflammatory status, thus limiting the damage associated with inflammatory insults of any nature.

Besides the activities attributed to the membrane-attached LRP1, LPS-triggered inflammation may also result in proteolytic processing of LRP1 and subsequent release of LRP1 derivates including secreted LRP1 and soluble LRP1 (sLRP1), which consists of the entire LRP1 α-chain and part of the β-chain ectodomain, in the plasma, where it regulates cell signaling and cytokine expression (55). The increase in sLRP1 was observed in mouse plasma following injection of LPS and in chronic inflammatory states such as rheumatoid arthritis (RA) and osteoarthritis (OA), suggesting that sLRP1 may represent a biomarker of inflammation. When sLRP1, purified from human plasma, or full-length LRP1, purified from mouse liver, was added to cultured macrophages, it resulted in a significant activation of NF-κB, JNK and p38 MAPK (55). The mechanisms associated with this apparent pro-inflammatory activity *in vitro* are not understood. In addition, another report has shown that sLRP1 promotes inflammation in microglial cells (56). A cell surface binding receptor for sLRP1 has not been identified and whether sLRP1 can act as a scavenger receptor is unknown. However, common ligands of LRP1 (A2MG, tPA and RAP) do not alter this pro-inflammatory effect of sLRP1. Furthermore, the *in vivo* activity of sLRP1 may differ from the *in vitro*, where cells are cultured with fetal serum, which may have ligands of sLRP1 not found in the adult. Yet, despite these reports, sLRP1 has been associated with anti-inflammatory signaling. sLRP1 was found to inhibit αMβ2 integrin-mediated adhesion of cells to fibrinogen, thus suggesting that sLRP1 may attenuate inflammation by modulating integrin function (57). sLRP1 also reduces the pro-inflammatory effects of TNF-a (revised in the next section).

A growing number of evidences implicates membrane-bound LRP1 in modulating the recruitment of circulating inflammatory cells to the injured site and their activity (41). In LPS-induced lung injury, LRP1 cooperated with bone morphogenic protein-binding endothelial regulator (BMPER) in the activation of pulmonary endothelium (58). After intratracheal or intraperitoneal LPS administration, migrating monocytes interact with β2 integrin to reach the alveolar space (59). In monocyte-derived macrophages (MDM), LPS induced LRP1 binding and endocytosis of αMβ2 integrin, suggesting that LRP1 may facilitate migration of activated macrophages, an essential step for resolution of acute inflammation (57). Inhibition or deletion of LRP1 in MDM enhanced NF-κB activation and release of IL-1β, TNFα, IL-6, CCL2, CCL3, CCL4, CCR5, and CXCL10. Of note, some of these factors (i.e., TNFα, IL-6, CCL2, CCL3, CXCL10) are strong mediators of neutrophil infiltration. Furthermore, LRP1-deficient MDM are more sensitive to LPS stimulation as they produce increased levels of pro-inflammatory mediators compared to LRP1-expressing MDM (60). In MDM and peritoneal macrophages, loss of LRP1 down-regulated M2 marker expression, while increasing macrophage response to M1 stimuli induced by poly(I:C), a TLR3-binding ligand (60). This finding indicates that LRP1 facilitates the M1/M2 conversion, promoting the development of an anti-inflammatory M2 functional phenotype at the site of injury (Figure 4B). Thus, LRP1 appears to control macrophage polarization and cytokine output, and consequently inhibit leukocyte infiltration (Figure 4C). In addition, the expression of LRP1 increases across differentiation and maturation of monocytic cells (61), suggesting that LRP1-mediated signaling may eventually contribute to the resolution of the active inflammatory response.

#### LRP1 as a Modulator of Tumor Necrosis Factor α (TNFα) Signaling

TNFα is one of the most pleiotropic cytokines described in mammals. Despite TNFα has been originally known for being a potent tumoricidal agent, it is an essential mediator of inflammation both directly and indirectly, through its ability to induce the expression of interleukin-6 (IL-6) (62). TNF-induced tumor necrosis factor receptor-1 (TNFR1) signaling generally results in the activation of the NF-κB and/or several additional death and non-death signaling pathways such as caspase-8, mitogen activated protein kinase (MAPK) and JNK (62). The LRP1 receptor interferes with the TNFα signaling pathway and modulates the inflammatory response (30).

Besides serving as a regulatory receptor by binding signaling adaptor proteins involved in inflammatory pathways, LRP1 exert an anti-inflammatory effect by regulating the expression of cell-surface receptors including tumor necrosis factor receptor 1 (TNFR1). In macrophages isolated from mice, LRP1 deficiency increased the activation of NF-κB pathway in response to TNFα, and the expression of inducible nitric oxide synthase (iNOS), IL-6, C1r, and monocyte chemoattractant protein-1 (MCP-1) (63). It was therefore proposed that LRP1 is capable of suppressing the production of inflammatory mediators both directly and indirectly, by down-regulating TNFR1-dependent cell signaling through the IκB kinase α (IKKα) - NF-κB pathway (63) (Figure 4A). Interestingly, exposure of cultured human lung fibroblasts to TNFα or bronchoalveolar lavage fluid (BALF) from ARDS patients induced the expression of membrane type-1 metalloproteinase (MT1-MMP) leading to increased shedding of LRP1, whereas the presence of a blocking antibody against TNFα reduced sLRP1 levels in the conditioned medium (64). In the context of atherosclerosis, loss of macrophage LRP1 conferred resistance to the TNFα-inhibitor adalimumab, and enhanced plaque cellularity and inflammation, suggesting that the anti-atherosclerotic effects of TNFα blockade may be dependent on the presence of macrophage LRP1 (65).

In the peripheral nervous system (PNS), Schwann cells (SCs) in an injured nerve displayed enhanced expression of LRP1 (66). LRP1 was reported to act as an early injury detector by binding proteins released in the earliest stages of PNS injury such as A2MG and tissue plasminogen activator (tPA) and, thereby, inducing c-Jun phosphorylation and/or ERK1/2 activation, and subsequent expression of inflammatory mediators (67). In contrast with membrane-bound LRP1, purified sLRP1 injected into mouse sciatic nerves reduced TNFα-induced p38 MAPK activation, decreased local expression of TNFα and IL-1β, and alleviated neuropathic pain in a model of chronic constriction injury (66). Furthermore, sLRP inhibited TNFα-induced activation of p38 MAPK and ERK/MAPK in cultured SCs, astrocytes, and microglia. Interestingly, in this model, the activity of sLRP did not involve TNFα binding, but rather glial cell preconditioning, so that the subsequent response to TNFα was inhibited, thus attenuating the inflammatory response (66).

Collectively, these evidences support the role of LRP1 as a suppressor of inflammation and cytokine release after injury by regulating several inflammatory pathways, including LPS and TNFα signaling cascades.

### LRP1 and Tissue Healing

Following the successful clearance of pathogens and potentially dangerous agents, the inflammation must be efficiently resolved to prevent collateral damage to the hosts' tissues. Fibrosis is the result of chronic inflammation induced by a variety of noxious stimuli (68). Growth factors, cytokines and chemokines produced by damaged cells stimulate the proliferation and recruitment of leukocytes across the ECM (68). Lymphocytes and other cells surrounding the injured site secrete pro-fibrotic cytokines and growth factors which further activate macrophages and fibroblasts. Activated fibroblasts synthetize collagen and differentiate into myofibroblasts, which promote wound contraction (68). However, an uncontrolled, unresolved inflammatory response triggers excessive ECM production, leading to the formation of an unfunctional fibrotic scar that precludes normal healing. The LRP1 receptor has the ability to modulate several molecules implicated in tissue fibrosis, by binding these molecules and regulating their plasma concentration, by coupling with their specific receptor kinases and/or by interacting with intracellular adaptor molecules and signaling factors through its cytoplasmic domain (30, 41).

#### LRP1 in TGF-β1 and CTGF Signaling

Among inflammatory and extracellular regulatory cytokines, TGF-β1 plays a central role as it possesses both immunomodulatory and fibrogenic characteristics. TGF-β1 mediates signals to the nucleus through cell surface transmembrane receptors with kinase activity and cytoplasmic effectors such as Smad proteins (68). LRP1 has been described as one of the cell surface receptors responsible of modulating TGF-β1 activity as described hereafter. In 2003, Huang and colleagues demonstrated that type V TGF-β receptor (TbetaR-V) is identical to LRP1 (69). Furthermore, evidences suggest that LRP1 is required for fibrotic responses mediated by TGF-β1. In rat kidney interstitial fibroblasts, the LRP1 ligand tPA promoted TGF-β1-mediated α-SMA and type I collagen expression by binding to LRP1 and inducing its rapid tyrosine phosphorylation (70). LRP1 in turn facilitated β1 integrin recruitment and downstream integrin-linked kinase (ILK) pathway activation (70). In the murine renal interstitium after obstructive injury, tPA and α-SMA colocalized with LRP1, and tPA deficiency reduced LRP1-mediated myofibroblast activation (70). In addition, binding of tPA to LRP1 promoted fibroblast survival through activation of the ERK1/2/p90RSK/Bad pathway and fibroblast proliferation via a mechanism involving ERK1/2, p90RSK, GSK3b, and Cyclin D1 (71). LRP1 was also identified as an endocytic receptor for decorin, a small leucine-rich proteoglycan already known to modulate the activity of TGF-β and other growth factors (72). Evidences showed that both LRP1 and decorin are necessary for TGF-β-dependent binding and signaling (72). In wild type myoblasts, inhibition of decorin binding to LRP1 or depletion of LRP1 reduced TGF-β response. The LRP1/decorin modulatory pathway also required Smad pathway activation by TGF-β and involved phosphatidylinositol 3-kinase (PI3K) (72). In a murine model of induced skeletal muscle damage, the expression of LRP1 and decorin was elevated concomitantly with TGF-β and CTGF (73). The pro-fibrotic molecule, connective tissue growth factor (CTGF), is selectively induced by TGF-β1 and these two cytokines are coordinately expressed at sites of tissue repair (74). Notably, in 2001, CTGF has been described as a ligand of LRP1 (75). In fibroblasts, incubation of CTGF in combination with TGF-β1 potentiated TGF-β1 effect, including myofibroblast activation, *de novo* expression of α-SMA, and extracellular accumulation of fibronectin (76). CTGF induced tyrosine phosphorylation of LRP1 intracellular domain and subsequent activation of ERK1/2 signaling, whereas the LRP1-antagonist, RAP, inhibited these effects (76).

**Figure 5:**
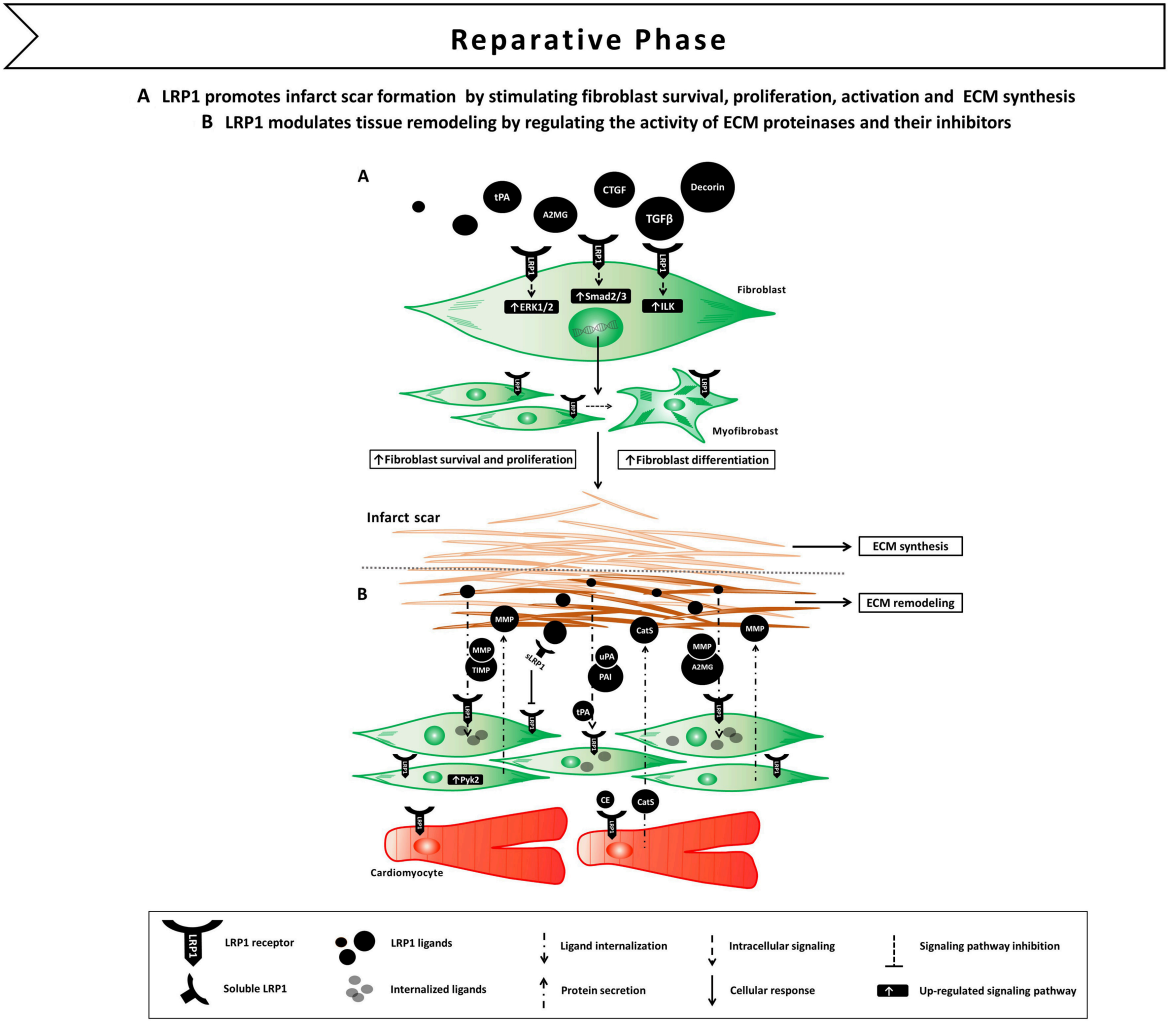
Proposed model of LRP1 involvement in the reparative phase following AMI. **(A)** LRP1-mediated signaling promotes fibroblast survival, proliferation and differentiation in myofibroblast. LRP1 appears to potentiate transforming growth factor β (TGF-β) and connective tissue growth factor (CTGF) signaling, thus facilitating extracellular matrix (ECM) deposition and scar formation. **(B)** LRP1 plays a major role in tissue remodeling as it serves as a functional receptor for ECM proteinases and their own inhibitors.

These experimental data indicate that activation of LRP1 signaling following tissue injury induces fibroblast survival, proliferation and activation leading to scar formation (Figure 5A). The fact that LRP1 modulates the activity of different pro-fibrotic molecules, such as TGF-β and CTGF, opens interesting opportunities of fine tune regulation of tissue repair and fibrosis through LRP1 (77).

### LRP1 and Tissue Remodeling

The ECM is a dynamic and intricate arrangement of collagens, glycoproteins, proteoglycans, and growth factors. Tissue remodeling is a complex process that occurs in both physiological and pathological conditions, characterized by dynamic quantitative and qualitative changes to the ECM (78). Several proteolytic enzymes are able to regulate the ECM turnover, including members of the MMP family and the serine proteases tPA and urokinase-type plasminogen activator (uPA) (78). The catalytic activity of these enzymes is finely regulated by a series of specific or nonspecific inhibitors such as tissue inhibitors of MMPs (TIMPs) and SERPINs (78). In this section, we will briefly recapitulate the endocytic/signaling functions of LRP1 in modulating extracellular activity of matrix proteinases (79).

LRP1 was reported to mediate the internalization and lysosomal degradation or recycling of tPA and uPA, either free or complexed to their inhibitor PAI (80). In addition to its effect on tPA and uPA, LRP1 has been also implicated in the regulation of the extracellular levels of MMP-2, MMP-9 and MMP-13 (81–85). In fibroblasts, LRP1 formed a co-receptor system with the matricellular protein thrombospondin (TSP-2) to mediate the internalization of proMMP-2/TIMP-2 complexes (82). On the contrary, proMMP-9/TIMP-1 directly interacted with LRP1 through the hemopexin domain of MMP-9 for LRP1-mediated endocytosis (84). Moreover, LRP1 may also recognize noncomplexed members of the TIMP family including TIMP-1, TIMP-2, and TIMP-3 via an MMP-independent mechanism to mediate their clearance (85).

Interestingly, matrix proteinases and their inhibitors have the ability to elicit LRP1-mediated signal transduction (79). Binding of A2MG or tPA to LRP1 induced LRP1 its phosphorylation and subsequent activation of downstream MAPK-ERK1/2, inducing MMP-9 expression and secretion (86, 87). More complexity is added by the fact that sLRP1, which is released during the inflammatory reaction (55), conserves the ability to bind matrix proteinases and inhibitors, and increase their extracellular half-life by preventing membrane LRP1-mediated clearance (79). Collectively, these results demonstrate that LRP1 is a functional receptor for matrix proteinases and their own inhibitors, and suggest a major role for LRP1 in modulating remodeling of the ECM by regulating matrix proteinase activity at different levels (Figure 5B).

Besides serving as a mechanical scaffold for the attachment and organization of cellular structures, ECM molecules induce intracellular signaling through multiple cell surface receptors, including LRP1. In response to chemical and biophysical changes in the ECM, the matricellular protein TSP was shown to interact with the LRP1/CRT co-receptor system and signal focal adhesion disassembly, a transition from a strong to an intermediate adhesive state, and facilitates cell motility and survival (88–90). LRP1 was also necessary for TSP/CRT-induced signaling as TSP failed to stimulate PI3K or ERK signaling in LRP1 deficient fibroblasts (90). These observations highlight a direct role for LRP1 in coupling extracellular microenvironment to intracellular signaling in response to ECM changes.

In summary, these experimental evidences may allow one to speculate that LRP1 serves as a common receptor of multiple ligands to mediate restraint of the inflammatory response in peripheral tissues after injury, and subsequently promote scar tissue formation and remodeling.

### LRP1 and Cell Survival

Balance between cell survival and death plays a fundamental role in the regulation of the inflammatory response to injury and its sequelae on tissue repair. In host defense, cell death may act in a protective manner as death of infected cells may reduce microbial infections and alert the host through danger signals and inflammatory mediators (91). At the same time, a complex and finely regulated signaling network is activated to promote survival of non-lethally damaged neighboring cells, a crucial step in limiting tissue injury (91). Numerous studies have demonstrated that LRP1-mediated signaling is responsible of regulating the cell fate by promoting survival and inhibiting death of the damaged yet salvageable cells within the injured tissue (21).

Elegant studies by Strickland's group have shown that LRP1 associates with the platelet-derived growth factor (PDGF) receptor-β in endosomal compartments and its cytoplasmic domain is rapidly tyrosine phosphorylated by Src family kinase (SFK) (92). PDGF-mediated phosphorylation resulted in increased association of the adaptor protein Shc with LRP1 at the second NPxY motif (92), and the LRP1/PDGFR-β co-receptor system activated the MAPK/ERK and PI3K-Akt pathways in mouse embryonic fibroblasts (93). Intriguingly, the MAPK/ERK and PI3K-Akt pathways are the two main pro-survival signaling cascades of the Reperfusion Injury Salvage Kinase (RISK) pathway, which confers powerful cardioprotection when specifically activated at the time of myocardial reperfusion (94).

In the CNS, the expression of LRP1 is critical for the survival of primary neurons under stress conditions such as trophic withdrawal, apoptosis inducers or amyloid-beta-induced neurotoxicity (95). Depletion of LRP1 through lentiviral short hairpin RNAs led to the activation of caspase-3 and increased neuronal apoptosis (95). Notably, LRP1 forebrain knock-out mice displayed decreased insulin receptor and phospho-Akt levels, suggesting that restoring LRP1-mediated signaling might be beneficial to inhibit cell death and neurodegeneration (95). Treatment of embryonic sensory neurons with structurally diverse LRP1 agonists, LRP1-receptor binding domain (RBD) of A2MG or hemopexin domain of MMP-9, led to a sustained activation of ERK1/2 and subsequently promoted cell survival and neurite extension, which were blocked by co-incubation with the LRP1 antagonist, RAP (96). Likewise, site directed mutagenesis of the LRP1 ligand, RBD, to preclude LRP1 binding failed to elicit these effects, confirming LRP1 specificity (96). Moreover, in a rat model of spinal cord injury, intrathecal infusion of the LRP1 agonists significantly increased sensory axonal regeneration and sprouting compared with control-infused animals (97).

The group of Gonias reported that binding of A2MG and tPA to neuronal LRP1 induced SFK-dependent transactivation of the Trk receptor and subsequent phosphorylation of Akt and ERK, which resulted in neurite outgrowth (98). Moreover, injection of the RBD of A2MG into rat dorsal root ganglia induced Trk receptor recruitment, which was inhibited by the LRP1 antagonist, RAP (98). The same group demonstrated that the second NPxY motif of LRP1 was also reported to interact with N-methyl-D-aspartate (NMDA) receptor through the postsynaptic density protein 95 (PSD95) and rapidly activate ERK1/2 following A2MG and tPA binding to LRP1 extracellular domain (99). Interestingly, the researchers demonstrated that the LRP1 ligand lactoferrin, functioning as a signaling antagonist, inhibited Trk receptor phosphorylation and ERK1/2 activation in response to tPA, suggesting that LRP1-initiated cell signaling may be partially ligand-specific (99). Ligand-specific co-receptor recruitment (i.e., PDGF, Trk and NMDA receptors) may therefore provide a mechanism by which LRP1 triggers different signaling responses including inhibition of programmed cell death and promotion of cell regeneration following injury.

By means of an LRP1 receptor chimera (sIgG-LRP) bearing the intracellular domain of human LRP1 fused to an extracellular IgG-F(c) portion, Lutz and colleagues demonstrated that, similar to the effects exerted by the naturally occurring LRP1 agonist A2MG on endogenous LRP1, sIgG-LRP modulated mitogen-induced Elk-1 and c-Jun transcriptional activity and conferred significant protection against apoptotic cell death (100). Mechanistically, sIgG-LRP co-localized with mitogen-induced c-Jun N-terminal kinase (JNK) at the plasma membrane compartment and, by sequestering JNK and inhibiting its nuclear translocation, sIgG-LRP inhibited JNK signaling pathway (100). Notably, the JNK pathway plays a role in apoptotic as well as non-apoptotic programmed cell death mechanisms including those of necroptosis, ferroptosis, pyroptosis, and autophagy (101). Therefore, further studies to determine the specific involvement of LRP1-mediated signaling in these processes are needed.

In PNS injury, stimulation of LRP1-mediated signaling through different LRP1 ligands had a comparable pro-survival effect to that seen in CNS. Disruption of LRP1-initiated signaling resulted in decreased activation of Akt, associated with increased caspase-3 levels in SCs (102). Similarly, SC-restricted LRP1 deletion worsened peripheral nerve injury (103). Treatment of cultured rat and human SCs with A2MG, tPA, or MMP-9 in turn induced the LRP1-mediated phosphorylation of c-Jun, a central step in the initiation of SC repair program, and the activation of Akt and ERK1/2, a crucial event in the promotion of SC survival (66, 102, 104). When the LRP1 agonists were injected into crush-injured rat sciatic nerves *in vivo*, the same effects were observed (66). The ability of LRP1 to bind proteins released in the earliest stages of injury and to induce c-Jun phosphorylation and Akt-ERK1/2 activation supports a model in which LRP1, on the one hand, functions as an early injury detector and, on the other, facilitates repair and survival of injured cells.

Collectively, these experimental evidences allow one to speculate that activation through LRP1 agonists of LRP1-mediated signaling following tissue injury represents a key mechanism promoting resolution of the inflammatory response and formation of a functional scar, while enhancing survival of the damaged yet salvageable cells. Based on its unique properties, it has been recently hypothesized that targeting LRP1 in the heart may induce cardioprotective signals following injury (20, 21).

## Role of LRP1 in the Ischemic Heart

The specific contribution of LRP1 to the cardiac homeostasis has not been appropriately investigated. This is complicated by the fact that LRP1 deficient mice are embryonic lethal and therefore in the past it has been difficult to study the homeostatic role of LRP1 in the heart (26). However, there is growing evidence demonstrating that LRP1 plays a central role in the physiological response of the heart to injury. The LRP1 receptor is basally expressed in the heart, and its levels significantly increase in response to stress or injury including exposure to apoptosis-inducers, lipid and glucose overload, and ischemia (105, 106). LRP1 is expressed in all the heart resident cellular types. *In vitro*, LPS and hypoxia strongly enhanced LRP1 mRNA and protein expression through the activation of HIF-1α in HL-1 cardiomyocytes and neonatal rat ventricular myocytes (NRVMs) (106), as well as in human VSMCs and endothelial cells (ECs) (107). *In vivo*, LRP1 was found to be markedly upregulated in the ischemic myocardial tissue in models of experimental AMI and in ischemic cardiomyopathy patients (20, 108, 109). After AMI, LRP1 was acutely expressed in cardiomyocytes, and in fibroblasts at later stages.

Some LRP1 agonists can modify the outcome of cardiomyocyte ischemia *in vitro* and *in vivo*. A2MG is an acute phase protein released into the serum following myocardial ischemia. Shortly after stimulation of rat ventricular cardiomyocytes with A2MG, LRP1 activated MEK1/2-ERK1/2 and PI3K-Akt, the two pro-survival signaling cascades as part of the RISK pathway (94), resulting in increased cell volume and protein synthesis (110). Furthermore, treatment with A2MG improved cardiomyocyte function as shown by augmented sarcoplasmic reticulum Ca^2+^-ATPase (SERCA-2) expression, diastolic and systolic [Ca^2+^] levels, and contractile responsiveness after electrical stimulation (110). Comparable effects were achieved upon treatment of cardiomyocytes with a stimulating LRP1 antibody and were counteracted upon coincubation with the LRP1 antagonist RAP, suggesting that activation of LRP1-initiated signaling through A2MG improves cardiac cell function *in vitro* (110) (Figure 3B).

In HL-1 cardiomyocytes, hypoxia increased the expression of LRP1 which in turn mediated the internalization of very low-density lipoprotein-cholesterol ester (VLDL-CE), whereas inhibition of LRP1 scavenging function through lentiviral-mediated interfering RNA prevented intracellular lipid accumulation (106). Similarly, LRP1 upregulation was accompanied by CE overaccumulation in the infarct areas as well as in the bordering myocardium in a porcine model of experimental AMI (108). While excessive LRP1-mediated hypoxia-induced fat internalization may be associated with cardiomyocyte dysfunction, nevertheless it might represent a compensatory mechanism by which the ischemic heart, through LRP1's scavenging function, reduces the oxidation of extracellular lipids and increases intracellular lipid levels to use as source of energy. Although LRP1 inhibition reduced VLDL-induced waves and irregular responses, lack of a functional LRP1 increased the percentage of inactive cardiomyocytes and worsened their resistance to calcium overload, indicating a pivotal role for LRP1 as a signal-transducing receptor that mobilizes calcium intracellular release (106). Hypoxia-induced overexpression of LRP1 led to increased levels of phosphorylated Ca^2+^-dependent nonreceptor protein tyrosine kinase (PTK) proline-rich tyrosine kinase 2 (pPyk2), which subsequently reduced, via HIF-1α overaccumulation, SERCA2 mRNA expression and the mean amplitude of calcium release from sarcoplasmic reticulum (111) (Figure 3B). Despite the negative effect of HIF-1α overexpression on SERCA2 depletion under hypoxic conditions, increased HIF-1α levels in the heart have been reported to protect against acute myocardial ischemia *in vivo* (112). Moreover, it was previously reported that LRP1 intracellular domain has the capacity to retain Ca^2+^, thus LRP1 might directly influence the intracellular availability of calcium (113). These evidences, although inconclusive, highlight a crucial role for LRP1 in coupling cardiomyocyte energy metabolism and contractile function.

Numerous studies have identified LRP1 as a key regulator of post-infarction remodeling through its scavenging/signaling functions in cardiac myocytes and fibroblasts, two of the most abundant cell types in the heart.

Studies from the group of Llorente-Cortés showed that LRP1 levels significantly increased in the peri-infarct and infarct areas at 10 and 21 days after permanent coronary ligation in mice (114). LRP1 colocalized with the fibroblast marker vimentin, indicating that LRP1 is mostly expressed by cardiac fibroblasts at this stage (114). Myocardial LRP1 also colocalized with pPyk2 and MMP-9, suggesting that LRP1/pPyk2/MMP-9 axis may play a role in ECM remodeling during the fibrotic post-infarction phase. *In vitro* experiments confirmed that hypoxia induced LRP1 overexpression together with increased Pyk2 phosphorylation and MMP-9 activity in cardiac fibroblasts (114) (Figure 5B). Interestingly, pERK1/2 expression was strongly upregulated at day 1 post-infarction, whereas pPyk2 expression peaked in the infarct areas at 10 and 21 days, suggesting that LRP1 mediates distinct spatial and temporal activation of ERK1/2 and Pyk2 in cardiomyocytes and fibroblasts, respectively (114). The differential spatio-temporal contribution of ERK1/2- and Pyk2-mediated pathways may explain the bifunctional role of LRP1 in regulating cardiac myocyte survival immediately after ischemia and cardiac remodeling in the later stages.

The LRP1/pPyk2 regulatory axis has been also implicated in hypoxia-induced vascular remodeling as revealed by the fact that silencing of LRP1 attenuated pPyk2 phosphorylation and MMP-9 activation, and mitigated human VSMC migration (115). However, others have shown that disruption of LRP1 by gene deletion or endogenous miR-205 overexpression in VSMCs may hinder the removal of pericellular MMP-9, leading to excess MMP-9 remaining in the extracellular matrix and disruption of vascular wall integrity (116). Interestingly, loss of a functional LRP1 protein favored higher expression and release of pro-inflammatory cytokines such as IL-1β, IL-6, and MCP-1 in hypoxic human VSMCs (115).

In an experimental model of non-reperfused AMI, LRP1 was scarcely detectable in the infarct areas in the inflammatory phase of remodeling after AMI, whose main cellular components are macrophages and neutrophils (114). It has been previously described that inflammatory mediators may reduce LRP1 expression in macrophages through sterol regulatory element-binding protein 1 (SREBP-1) (117), nevertheless, lack of LRP1 exacerbated the inflammatory response, as macrophage lacking LRP1 displayed increased TNF-α, MCP-1, and MMP-9 production (45). It is therefore unclear whether the low LRP1 levels in inflammatory cells at day 1 after AMI observed in this experimental model of permanent coronary ligation are a cause of inflammation, a consequence or both (114). Myocardial LRP1 was significantly upregulated in the ischemic areas during the fibrotic phase of remodeling after AMI together with increases in cardiac fibroblast proliferation (114). Previous studies showed that LRP1 in fibroblasts is necessary for expression of TGF-β (72), a crucial inducer of the specialized myofibroblast-like phenotype that fibroblasts adopt in response to injury. Therefore, hypoxia-induced LRP1 overexpression in cardiac fibroblasts may promote TGF-β signaling (118). These evidences indicate that LRP1 plays a major role in the regulation of TGF-β signaling and MMP-9 activity in cardiac fibroblast after AMI, and highlight the potential role of LRP1 modulation for treatment of cardiac remodeling (Figure 5).

### Role of LRP1 in Myocardial Ischemia and Reperfusion Injury

The binding of protease-inhibitor complexes to LRP1 is seen across the entire spectrum of SERPINs (16, 119) and it has been previously described to inhibit the inflammatory response (43, 44, 60, 66), and induce a cytoprotective effect through phosphorylation of Akt and ERK1/2 protein kinases (66, 96, 98, 102, 104). Recently, different members of the SERPIN superfamily, including AAT, A2MG, and AT_III_, when administered upon reperfusion, have been shown to reduce infarct size, improve systolic contractility and ameliorate cardiac remodeling by eliciting powerful pro-survival and anti-inflammatory signals in the ischemic myocardium (120–124).

In a model of reperfused AMI, AAT-treated mice exhibited a significantly reduced infarct size and a preserved LV ejection fraction in the initial (24 h) and late (7 days) phases after AMI, together with a powerful reduction in caspase-1 activity (120). AAT similarly reduced cardiomyocyte death and caspase-1 activity after LPS stimulation or simulated ischemia *in vitro* (120). The anti-inflammatory effect of AAT was confirmed by the significant reduction in both pro-inflammatory cytokines levels and leukocytes infiltrating the heart tissue after ischemia-reperfusion (120). Likewise, plasma-derived AAT considerably reduced the acute myocardial inflammatory injury after ischemia-reperfusion in the mouse leading to preservation of viable myocardium and systolic function, and the effects persisted across a wide range of experiments in the mouse reproducing clinical relevant scenarios, such as variable duration of ischemia (up to 75 min), delay in administration of the drug (up to 30 min), and a large therapeutic index (121). The protective effects of AAT have been demonstrated to be independent from its neutrophil elastase-inhibiting activity, as a recombinant protein composed of human AAT fused to the human immunoglobulin (Ig) G1 Fc fragment (rhAAT-Fc) inhibited the inflammatory injury following acute ischemia (124). Notably, treatment with an LRP1 blocking antibody vanished the benefits of AAT, indicating that the cardioprotective effect elicited by AAT is mediated by LRP1 signaling (20). Administration upon reperfusion of plasma-derived A2MG also elicited cardioprotective signals across a large dose range (122). Likewise, AAT_III_, another plasma SERPIN known to bind LRP1, limited infarct size independently of its anticoagulant activity after experimental AMI in mice (123). It was further reported by the same authors that AT_III_ up-regulated the release of prostacyclin in the ischemic myocardium, and inhibited the expression of pro-inflammatory cytokines TNF-α and IL-6 *in vivo* by attenuating ischemia/reperfusion-induced JNK and NF-κB signaling pathways (123) (Figure 4A).

The data from these preclinical animal studies indicate that distinct non-selective LRP1 agonists derived from SERPINs, including AAT, A2MG, and AAT_III_ induce a similar cardioprotective effect, independent of their ability to inhibit proteases. Therefore, it was recently hypothesized that selectively targeting LRP1 by means of a selective synthetic agonist would provide a comparable cardioprotective effect (20). During experimental AMI, LRP1 activation with a synthetic peptide agonist of LRP1 (SP16) given intraperitoneally at reperfusion led to a powerful cardioprotective signal reducing infarct size and cardiac systolic dysfunction in a dose-dependent manner (20). In contrast, inhibition of LRP1 with a blocking antibody abolished the benefits of SP16, hence confirming that LRP1 is essential to protect the heart from acute ischemia (20). Furthermore, cardiomyocyte-restricted LRP1 deletion abolished the cardioprotective effects of SP16, confirming the specific role of cardiomyocyte LRP1 signaling in the cardioprotective mechanism [Personal communication by the authors]. As measures of the pro-survival signal of the LRP1 agonist, pAkt levels increased together with a significant reduction in in proapoptotic to antiapoptotic Bac/Bcl2 ratio and caspase-3 expression in the myocardial tissue at 24 h from acute ischemia, suggesting that LRP1 rapidly activates the RISK pathway upon reperfusion (20) (Figure 3B). *In vitro*, the SP16 peptide significantly inhibited LPS-induced NF-κB activation and NLRP3 inflammasome formation in monocytes (20). When tested in a mouse model of endotoxemia, SP16 significantly reduced mortality, demonstrating the anti-inflammatory activity of the LRP1 agonist *in vivo* (20). These findings are consistent with the marked reduction in infarct scar size and leukocyte infiltrating the peri-infarct area observed at 7 days from experimental AMI (20) (Figure 4C). Thus, stimulation of LRP1 signaling through a targeted agonist provided powerful anti-inflammatory and pro-survival signals, thus limiting the inflammatory injury that follows ischemia-reperfusion.

## Role of LRP1 in the non-ischemic Heart

The group of Llorente-Cortés observed that LRP1-mediated cardiomyocyte CE overaccumulation altered the structural and physical characteristic of tropoelastin (TE), one of the main components of ECM, by upregulating cathepsin S (CatS), a cysteine protease with the ability to degrade TE (105) (Figure 5B). The modulation of LRP1-mediated lipid-scavenging function in cardiomyocytes was therefore proposed to impact pathological ventricular remodeling associated with insulin-resistance and combined hyperlipoproteinemia, two conditions characterized by increased CE-rich lipoprotein concentrations (105).

Accumulating evidence shows that LRP1, together with CRT, mediates the beneficial actions of adiponectin on the cardiovascular system. Adiponectin is a fat-derived, plasma protein, down-regulated in obesity, that exerts cardioprotective effects in obesity-linked diseases and other diseases (e.g., doxorubicin [DOX] cardiac toxicity) (125). In cultured NRVMs, adiponectin stimulated Akt phosphorylation and inhibited DOX-induced cell death, whereas interference of the LRP1/CRT co-receptor system by siRNA or blocking antibodies inhibited the stimulatory effects of adiponectin on Akt activation and cardiomyocyte survival (125), suggesting that adiponectin-triggered LRP1-mediated signaling protects against DOX-induced cardiotoxicity. Besides its protective effect on cardiomyocytes, adiponectin protein increased cyclooxygenase-2 (COX-2) expression in cultured ECs, together with EC migration, differentiation and survival, thus promoting revascularization of ischemic muscle in a mouse hind limb model of vascular insufficiency (126) (Figure 3C). Notably, ablation of LRP1 abrogated adiponectin-stimulated COX-2 expression and endothelial responses (126). Obesity-related diseases are strongly associated with increased microvascular rarefaction, reduced collateralization in ischemic tissues and perivascular inflammation, which contributes to augmented vulnerability to ischemic insults. These data provide evidence that, under ischemic conditions, adiponectin improves endothelial function through LRP1/CRT-mediated increases in COX-2 signaling, and implicate this regulatory axis in the pathogenesis of obesity-related vascular diseases (126).

Numerous studies demonstrated that LRP1 controls lipid and glucose metabolism in liver, vasculature and adipose tissue, and it is also implicated in the regulation of leptin signaling and food intake in CNS (127). However, the role of LRP1 in regulating energy homeostasis in the ischemic heart has not been investigated until recently. In mouse cardiac-derived ECs, the intracellular domain of LRP1 interacted with the nuclear receptor PPARγ, a key regulator lipid and glucose metabolism, and positively regulated its transcriptional activity, which was potentiated upon treatment with the PPARγ agonist pioglitazone (128) (Figure 3C). As expected, depletion of endothelial LRP1 inhibited PPARγ activity and led to dysregulation of PPARγ lipid-handling target genes (128). These results indicate a complex functional role for endothelial LRP1 in maintaining systemic energy homeostasis.

## Therapeutic Potential of LRP1 Agonists for AMI

Clinical experience with LRP1 agonists is today limited to a clinical feasibility trial (VCU-α1RT pilot study, clinicaltrials.gov, NCT01936896) with AAT, a naturally occurring non-selective LRP1 agonist (129, 130), and a phase I clinical trial (clinicaltrials.gov, NCT03651089) with SP16, a synthetic selective LRP1 agonist (131). A single intravenous administration plasma-derived AAT within 12 h of admission and following standard-of-care treatment in patients with ST segment elevation acute myocardial infarction (STEMI) was well-tolerated and showed no treatment-related serious adverse events (129). CRP levels and estimated infarct size were significantly lower 14 days after admission in the AAT-treated group compared to a historical placebo-treated control group, indicating that plasma-derived AAT is associated with a blunted acute inflammatory response after AMI (129, 130). Furthermore, among the patients who received AAT, none experienced HF at 1 year. In contrast, 1 patient died and 9 patients (50%) had incident HF at 1 year in the non-AAT deficient comparison group (129, 130). In a randomized, double-blind, placebo-controlled phase I clinical trial testing SP16 in healthy volunteers, a single subcutaneous administration of the selective LRP1 agonist showed a favorable safety and tolerability profile (131).

Despite therapeutic advances, AMI remains a leading cause of morbidity and mortality worldwide (1). Coronary artery reperfusion is successful in shortening the duration of ischemia and reducing infarct size, however one potential limitation of the current paradigm is the lack of effective therapies to prevent reperfusion-mediated injury (4, 5, 11, 12). Experimental evidences suggest that this process accounts for up to 50% of the final infarct size, making it a potential target for cardioprotection (4). Prevention of mitochondria-mediated events with cyclosporine and other mitochondrial membrane permeability inhibitors has shown potential benefits in animal studies, yet provided disappointing results in clinical trials possibly because treatment is only effective if administered before ischemia or reperfusion, thus limiting the clinical translational feasibility of these strategies due to their narrow therapeutic window (132). Of note, the data with distinct LRP1 agonists showed that the cardioprotective effect is maintained up to 2 h after reperfusion in the mouse and within 12 h after reperfusion in patients (20, 120, 121, 129, 130). The gap between pre-clinical and clinical studies may also recognize other explanations such as poor experimental design, species differences, use of young and healthy animal models, and insufficient pre-clinical testing (12). Further research is therefore warranted to better elucidate the cellular and molecular mechanisms driving LRP1-mediated infarct-sparing effect, including conditional cell-specific LRP1 knockout, to determine the cell-specific role of LRP1 signaling in AMI, and testing the effectiveness of LRP1 agonism in the presence of different comorbidities.

## Conclusion

The inflammatory response following injury of any nature, while representing an attempt to promote healing, it may, itself, result in greater injury. The activation of an exaggerated inflammatory response in the heart following ischemic and non-ischemic injury leads to adverse cardiac remodeling and HF. Low-density lipoprotein receptor-related protein 1 (LRP1) is a ubiquitous endocytic cell membrane receptor with the ability to internalize a wide range of structurally and functionally diverse ligands. Furthermore, binding of certain ligands to LRP1 induces multiple intracellular signaling pathways capable of modulating the inflammatory reaction following organ injury, promoting tissue healing and cell survival in a ligand- and cytotype-specific manner. In preclinical studies, activation of LRP1-mediated signaling in the heart with non-selective and selective LRP1 agonists is associated with a powerful cardioprotective effect, reducing infarct size and cardiac dysfunction after AMI. The data from early phase clinical studies with plasma-derived α1-antitrypsin (AAT), an endogenous LRP1 agonist, and SP16 peptide, a synthetic LRP1 agonist, are encouraging and suggest that LRP1 modulation is safe. Activation of LRP1-mediated signaling through specific and non-specific LRP1 agonists may therefore represent a novel therapeutic strategy to reduce cardiac inflammatory injury, regulate infarct healing and promote cardiac recovery following AMI.

## Author Contributions

NP, MDB, AGM, AA, and ST have made substantial, direct, and intellectual contributions to the work and approved it for publication. NP wrote the initial draft of the manuscript, tables, and figures. MDB, AGM, AA, and ST critically revised the whole manuscript.

### Conflict of Interest Statement

AA has served as a consultant to Serpin Pharma (Manassas, VA, USA). ST has received an investigator initiated sponsored study supported by Serpin Pharma. The remaining authors declare that the research was conducted in the absence of any commercial or financial relationships that could be construed as a potential conflict of interest.
